# MPTP-Treated Zebrafish Recapitulate ‘Late-Stage’ Parkinson’s-like Cognitive Decline

**DOI:** 10.3390/toxics10020069

**Published:** 2022-02-04

**Authors:** Alim A. O. Bashirzade, Sergey V. Cheresiz, Alisa S. Belova, Alexey V. Drobkov, Anastasiia D. Korotaeva, Soheil Azizi-Arani, Amirhossein Azimirad, Eric Odle, Emma-Yanina V. Gild, Oleg V. Ardashov, Konstantin P. Volcho, Dmitrii V. Bozhko, Vladislav O. Myrov, Sofia M. Kolchanova, Aleksander I. Polovian, Georgii K. Galumov, Nariman F. Salakhutdinov, Tamara G. Amstislavskaya, Allan V. Kalueff

**Affiliations:** 1 Scientific Research Institute of Neuroscience and Medicine, 630090 Novosibirsk, Russia; cheresiz@yandex.ru (S.V.C.); belovaas@physiol.ru (A.S.B.); amstislavskayatg@physiol.ru (T.G.A.); 2 Institute of Medicine and Psychology, Novosibirsk State University, 630117 Novosibirsk, Russia; abkommem@gmail.com (A.V.D.); a.korotaeva2@g.nsu.ru (A.D.K.); soheil.aziziarani@gmail.com (S.A.-A.); Azimirad@mail.ru (A.A.); odle.eric1@gmail.com (E.O.); emmagild@gmail.com (E.-Y.V.G.); 3 Vorozhtsov Novosibirsk Institute of Organic Chemistry SB RAS, 630090 Novosibirsk, Russia; ardashov@nioch.nsc.ru (O.V.A.); volcho@nioch.nsc.ru (K.P.V.); anvar@nioch.nsc.ru (N.F.S.); 4 ZebraML, Inc., Houston, TX 77043, USA; dmitry.bozhko@zebraml.com (D.V.B.); vladislav.myrov@zebraml.com (V.O.M.); sofiia.kolchanova@zebraml.com (S.M.K.); alex.polovian@zebraml.com (A.I.P.); georgy.galumov@zebraml.com (G.K.G.); 5 Ural Federal University, 620002 Yekaterinburg, Russia; 6 Neurobiology Program, Sirius University of Science and Technology, 354340 Sochi, Russia; 7 Moscow Institute of Physics and Technology, 141701 Moscow, Russia; 8 Granov Scientific Research Center of Radiology and Surgical Technologies, 197758 St. Petersburg, Russia; 9 Institute of Experimental Medicine, Almazov National Medical Research Centre, 197341 St. Petersburg, Russia; 10 Institute of Translational Biomedicine, St. Petersburg State University, 199034 St. Petersburg, Russia; 11 School of Pharmacy, Southwest University, Chongqing 400715, China

**Keywords:** 1-Methyl-4-phenyl-1,2,3,6-tetrahydropyridine (MPTP), zebrafish, Parkinson’s disease, inhibitory avoidance task, spontaneous alternation, artificial intelligence

## Abstract

The zebrafish is a promising model species in biomedical research, including neurotoxicology and neuroactive drug screening. 1-Methyl-4-phenyl-1,2,3,6-tetrahydropyridine (MPTP) evokes degeneration of dopaminergic neurons and is commonly used to model Parkinson’s disease (PD) in laboratory animals, including zebrafish. However, cognitive phenotypes in MPTP-evoked experimental PD models remain poorly understood. Here, we established an LD_50_ (292 mg/kg) for intraperitoneal MPTP administration in adult zebrafish, and report impaired spatial working memory (poorer spontaneous alternation in the Y-maze) in a PD model utilizing fish treated with 200 µg of this agent. In addition to conventional behavioral analyses, we also employed artificial intelligence (AI)-based approaches to independently and without bias characterize MPTP effects on zebrafish behavior during the Y-maze test. These analyses yielded a distinct cluster for 200-μg MPTP (vs. other) groups, suggesting that high-dose MPTP produced distinct, computationally detectable patterns of zebrafish swimming. Collectively, these findings support MPTP treatment in adult zebrafish as a late-stage experimental PD model with overt cognitive phenotypes.

## 1. Introduction

Parkinson’s disease (PD) is a highly prevalent and severely debilitating age-related neurodegenerative disorder [1] that typically manifests as progressive worsening of voluntary movements with cognitive and emotional deficits [2]. PD is characterized by the loss of nigral dopaminergic neurons that induce motor deficits (e.g., tremor, bradykinesia, rigidity and postural instability) [3], later also causing overt cognitive decline, including executive dysfunction, cognitive inflexibility and poorer working memory [4,5].

1-Methyl-4-phenyl-1,2,3,6-tetrahydropyridine (MPTP) is an agent that chemically ablates nigral dopaminergic neurons (SNpc) and, therefore, mimics clinical PD [6,7,8]. MPTP exposure is a well-established animal PD model [9], whose advantage (over other drugs and toxins) is in selective targeting SNpc [10,11]. Although MPTP itself is not neurotoxic, neuroglial cells metabolize it into the neurotoxic 1-methyl-4-phenylpyridinium (MPP+) cation [12,13]. Taken up by the dopamine transporter into dopaminergic neurons, MPP+ damages the mitochondrial oxidative phosphorylation system [14], affecting the ability of dopaminergic neurons to sustain activity and, hence, causing their death [15].

In addition to rodent MPTP-based models of PD [16], the drug is widely used to recapitulate PD in zebrafish, *Danio rerio* [17]. These aquatic PD models are particularly promising, given high genetic and physiological homology of zebrafish with humans, body transparency [18], and structural similarity between the zebrafish dopaminergic system and human striatum [19]. PD-like locomotor deficits are also well-reported in zebrafish models, observed as shorter distance swum, slower swimming, and frequent freezing bouts [20,21]. Although PD-like motor deficits caused by MPTP have been previously reported in zebrafish [22,23], their potential cognitive phenotypes in this model have not yet been tested.

To address this knowledge gap, here we applied an MPTP model to characterize PD-like cognitive deficits in adult zebrafish by assessing their spatial working memory and spontaneous alternation behavior (SAB) [24,25]. The innate drive to alternate locomotion is an important exploratory strategy in various species (from humans to fish), requiring good working memory capability [24]. The two commonly used types of SAB assays include the two-trial and the continuous SAB tests [25]. The latter assays have several clear practical advantages, as they are simpler to perform and require less handling of the experimental animals. To further reinforce our analyses, we also applied the artificial intelligence (AI)-based convolution neural networks to analyze SAB data obtained in the aquatic Y-maze test in MPTP-treated zebrafish.

## 2. Materials and Methods

### 2.1. Animals and Housing

A total of 1000 adult (~1 year old, ~1 g body weight) zebrafish of the wild type outbred long-fin strain (~1:1 male-to-female ratio) were acquired commercially from a local supplier (Pets, Novosibirsk, Russia). All fish were experimentally naïve and housed for at least 2 months in a 100-L holding tank, filled with system water maintained at 25 ± 1 °C and pH of 7.0 ± 0.1. The holding tank was equipped with a standard air pump (Champion CX-0098, 950 L/h, Chuangxing Electric Appliances Co Ltd., Zhongshan, China), water pump (Chosen CX-300, Chuangxing Electric Appliances Co Ltd.), and biological filters under constant aeration and filtration. Illumination was provided via fluorescent light tubes (140–160 lx) simulating a 14:10 h day/night cycle (lights on at 9:00 a.m.). All fish were fed once a day using commercial flake food. All procedures were performed according to the standards of zebrafish care [26]. All experiments were approved by the local ethics committee of the Scientific Research Institute of Neuroscience and Medicine (SRINM, Protocol 1/2021) and fully complied with National and International guidelines on humane animal experimentation. The outbred population selection for the present study was based on population validity considerations and their relevance for the present study. Briefly, although genetically controlled models (e.g., inbred zebrafish strains) can be a better reproducible and more reliable system for neurogenetics research, modeling CNS disorders, such as in the present study, involves ‘real’ human disorders affecting genetically heterogenous populations [27]. Thus, using outbred populations of zebrafish (such as selected here) is a more populationally valid and translationally relevant approach for the purpose of this study [28]. Using both sexes of zebrafish was chosen in the present study in order to more fully mimic heterogenous human populations.

### 2.2. Drug Treatment

MPTP was synthesized at the Division of Medicinal Chemistry of Vorozhtsov Novosibirsk Institute of Organic Chemistry (Siberian Branch, RAS, Novosibirsk, Russia), dissolved in dimethyl sulfoxide (DMSO) to obtain the 200-mg/mL (1.16-M) stock solution, aliquoted and stored frozen at −20 °C. The working MPTP solutions for different fish/dose groups were prepared on the day of the experiment by diluting the stock MPTP solution by phosphate buffered saline (PBS) to the appropriate concentration for each zebrafish study group, with the injection volume of 100 µL administered intraperitoneally per 1-g fish. All working MPTP solutions were adjusted to contain 1% of DMSO (% *v*/*v*), known to be devoid of behavioral or toxic effects in zebrafish.

### 2.3. Experimental Design and Acute MPTP Toxicity (LD_50_) Assay

At the beginning of the experiments, the mean fish weight (g) and its standard deviation (SD) were determined in a group of 25 fish randomly collected from the holding tanks, yielding the average body weight of 0.995 ± 0.09 g for individual fish in the present study, approximated to ~1 g and used for further calculations.

Zebrafish were next divided into five experimental groups (12–41 fish per group), each receiving a 100-µL intraperitoneal injection of 0, 50, 100, 200, and 400 µg MPTP in 1% DMSO/PBS (Supplementary Material S1). Fish locomotion was assessed 24 h after the drug exposure, in order to evaluate motor effects of MPTP (Supplementary Material S1). Assessing SAB, the Y-maze test was performed twice in this study, 6 and 24 h after MPTP administration. The inhibitory avoidance testing was performed for the next two days, twice daily. Acute toxicity/mortality was assayed by counting the number of fish dead 24 h post-injection, divided by the total number of fish per group, and expressed as the percentage of total. The lethality in the control group was further deduced from the obtained lethality in each experimental group, and the resulting values were plotted as the lethality curve. LD_50_ values were calculated using the linear regression of the constructed curves, based on the graphical method of Miller and Tainter [29]. The reference doses of MPTP were expressed as mg/kg body weight, based on an average fish body weight assessed (1 g) in the experimental colony.

### 2.4. Spontaneous Alternation and Inhibitory Avoidance Assays

**Figure 1:**
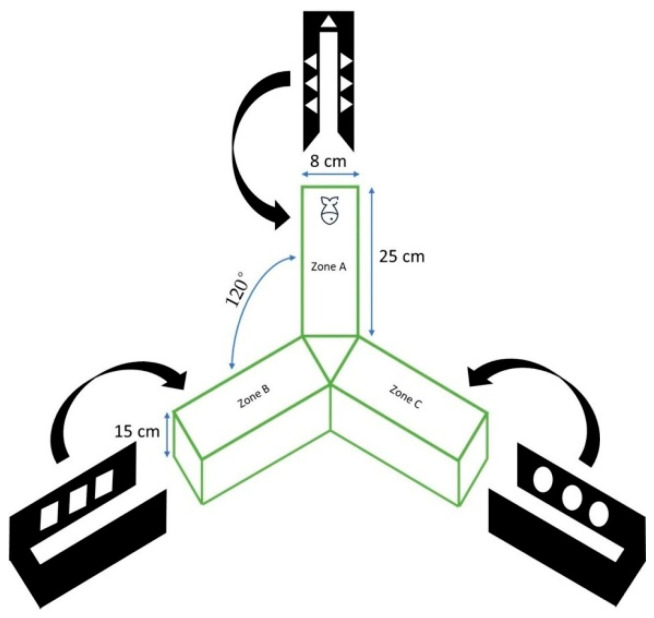
Schematic representation of Y maze apparatus, as in [30], with minor modifications. Removable arms were constantly swapped around.

The Y-maze test was performed 6 h and 24 h after MPTP administration, and used a Y-maze apparatus, according to [30], representing a Y-shaped enclosure with three arms (25 long × 8 wide × 15 high, cm, oriented at 120°), constructed from transparent glass (Figure 1). The inner surface of each arm was labeled with distinct visual cues (white geometric shapes) visible to the fish. Externally, the arms were covered by a black, opaque plastic cover. The maze floor was illuminated by an LED panel (130–150 lx), and the apparatus was filled with 3 L of fresh system water prior to each trial. Starting from a uniform initial position, fish were allowed to freely swim for 10 min while their behavior was recorded by a c922 Pro Stream digital camera (Logitech International S.A., Lausanne, Switzerland) positioned above the apparatus. Spontaneous alternation behavior (SAB) was calculated as a percentage of observed spontaneous alternations: SAB % = (the number of spontaneous alternations)/(total number of arms crossed – 2) × 100 %. Positional data were then extracted from the video files using the EthoVision XT-10 software (Noldus IT, Wageningen, The Netherlands) and further analyzed to calculate the number of inter-arm transitions.

The inhibitory avoidance test (IAT) used here represented a plexiglass box (30 long × 17 wide × 13 tall, cm) divided by a remote-controlled sliding divider into shallow and deep zones (3- and 11-cm deep, respectively). Two metal plates (16 × 10 cm) were fixed on the opposing walls of the deep zone and connected to a DC current generator (40 V, 100-ms pulse duration, 1-ms pulse delay, and 10-Hz pulse frequency). Videos were recorded on a Logitech c922 Pro Stream digital webcam positioned above the chamber prior to analysis in the EthoVision XT-10 software. The IAT experiment involved training and testing phases performed 24 h apart. During the training phase, fish were individually transferred to the shallow zone (with the closed divider) and allowed to swim for 1 min to acclimate. After the acclimation period, the divider was then opened remotely. In general, zebrafish prefer deep over shallow areas, and promptly retreat into a deeper zone when given the choice. The divider was remotely closed following the deep zone entry, after which the electric shock was delivered.

The testing phase commenced 24 h later, during which the fish were placed individually into the shallow zone (with the closed divider) for a 1-min acclimation, as in the training phase described above, but without the electric shock delivered upon the deep zone entry. For both training and testing IAT phases, the time difference between opening the divider and the initial deep zone entry was measured. Three non-avoiding fish were excluded from our analyses as they failed to enter the deep zone within 180 s.

### 2.5. AI-Based Analysis

Using neural networks is essential for collecting and interpreting unstructured research data [31]. A convolution neural network (CNN) sequentially applies convolution operators to an input and, thus, extracts the higher-level features, from basic lines and gradients to shapes [32]. Here, we used the ResNet34 CNN architecture, which offers the best balance between training time, complexity and the prediction quality [33], and has already been successfully applied to analyzing zebrafish behavioral data [34]. In the present study, we trained the computational model to predict MPTP concentrations using video recordings of SAB in fish in the Y-maze test. We extracted a fish position from the videos during the test using the EthoVision XT-10 software, cut each locomotor track into short 30-s frames, converted each track to an image, and used those images to train a neural network, similar to [34]. 

To assess the prediction validity of this model, we calculated its prediction accuracy as the percentage of correctly predicted classes across the hold-out validation dataset. We also aggregated predictions to compute a confusion matrix (N × M size, where N is the number of classes in the training dataset, M is the validation dataset, and the i,j cell value shows the fraction of samples that were predicted as class i, but belonged to class j) [35]. The computed prediction accuracies were aggregated across classes and used as a metric to construct a similarity network. This network was analysed using the Louvain algorithm, which assigns a community to each node based on the optimal data modularity. The method was chosen for its high-quality detection of distinct data clusters and fast data processing speed (see [34] for details).

We assumed that the effect of MPTP concentrations may vary between the two days of recording and therefore assigned a unique label for each combination of day and MPTP concentration of interest. To investigate any possible side effects, we performed multiple sets of computational experiments, first combining all classes into a single dataset to train a network to distinguish them, and next training the network on data from Day 1 to predict a drug effect based on Day 2 recordings (Table 1).

Prediction results are usually described as a confusion matrix—a square matrix where each row represents actual classes, and columns reflect the predicted classes. To assess significance of the model predictions, a permutation test shuffled all class labels and computed a baseline accuracy—a minimum level to consider prediction statistically ‘significant’, as in [34]. We then constructed a similarity graph using the confusion matrix as a matrix of edge weights and detected communities (small subsets of the graph nodes that share similar properties). Finally, we used the Louvain community detection method, as it yields optimal partitions [36], and has already been successfully used for zebrafish behavioral analyses [34].

### 2.6. Data Analysis

Data were analyzed using STATISTICA 10 software (TIBCO Software Inc., Palo Alto, CA, USA). Data were expressed as mean ± SEM. Normal distribution of data was assessed with Shapiro-Wilk’s W test. Parametrical variables were analyzed with one-way analyses of variance (ANOVA) followed by the LSD post-hoc test for significant ANOVA data. Non-parametrical variables were analyzed using the Kruskal–Wallis H test, where applicable. Correlations were calculated using the Pearson correlation coefficient. Dependent pair variables were analyzed by Student’s *t*-test. Pearson correlation was also performed on motor and cognitive data reported in Y-maze experiments here. The value of *p* was set at <0.05 in all analyses in the present study.

## 3. Results

### 3.1. Acute MPTP Toxicity in Adult Zebrafish (LD_50_ Assay)

**Figure 2:**
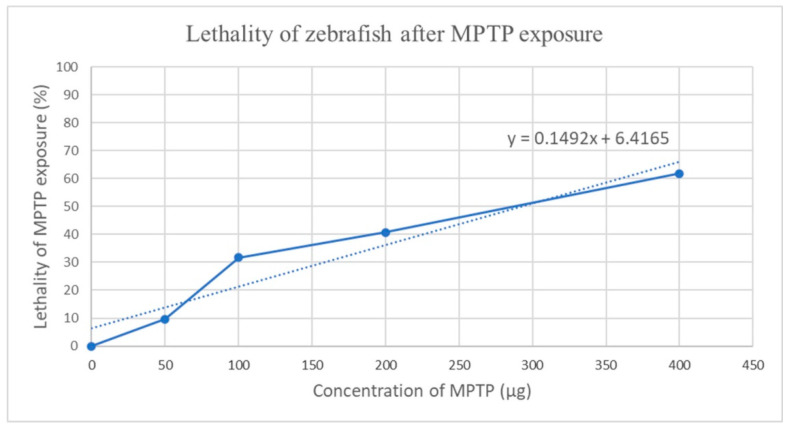
LD_50_ values calculated using the linear regression of the constructed curves, based on the graphical method of Miller and Tainter in the Excel software.

The lethality of escalating MPTP doses (0, 50, 100, 200, and 400 μg/1-g fish, expressed as mg/kg of body weight) were used to build the mortality curves and to determine LD_50_ values for adult zebrafish exposed to MPTP intraperitoneally (Figure 2). We estimated LD_50_ as 292 mg/kg, based on equations derived from the mortality curves.

### 3.2. Cognitive Behaviors

**Figure 3:**
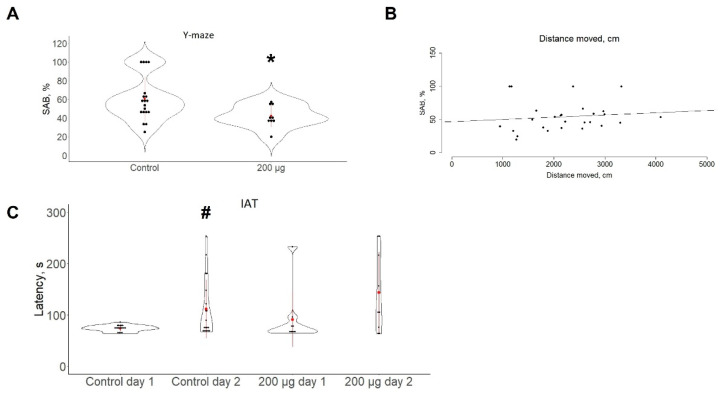
Analysis of cognitive functions in adult zebrafish in the aquatic Y-maze test (panel (**A**), assessed as % of spontaneous alternation behavior, SAB, *n* = 9–19 per group, analyzed using the unpaired two-sample *t*-test and Pearson correlation (Panel (**B**)) and the inhibitory avoidance test (IAT, *n* = 9–19 per group, panel (**C**), analyzed using paired sample *t*-test). Data are presented as the violin and dot plots. Red dots with lines represent mean ± SD; * *p* < 0.05 vs. control fish, unpaired two-sample *t*-test, # *p* < 0.05 vs. control day 1, paired *t*-test.

Zebrafish cognitive phenotypes in the present study were evaluated in control and 200-μg groups, based on AI analyses of locomotion, deeming this dose as most effective (see further results). SAB decreased in the 200-μg group compared to control fish (t(26) = 2.17, *p* < 0.04, Figure 3A). Moreover, correlational analyses of motor and cognitive data confirmed that the SAB rates were not accompanied by locomotor deficits (Pearson’s correlation r (26) = 0.12; *p* > 0.05, Figure 3B). Finally, the IAT assay revealed a significant difference in the latency to enter the trained deep zone between the two groups (t(18) = −2.92, *p* < 0.01, Figure 3C), successfully reproducing unaltered fear learning in the wild-type zebrafish.

### 3.3. AI-Based Analyses

**Figure 4:**
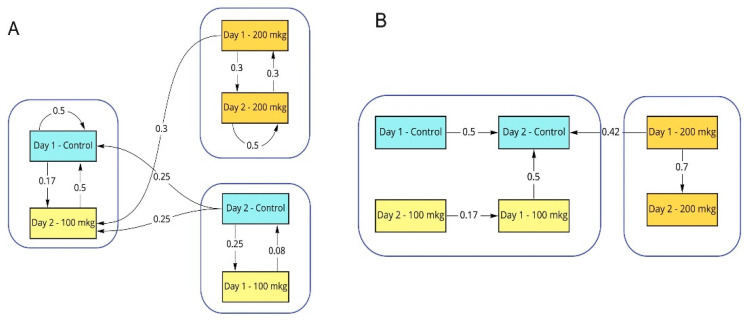
Artificial intelligence (AI)-based convolution neural network (CNN) used for analyses in two computational experiments involving a combined dataset (Panel (**A**)) and cross-day comparison (Panel (**B**)). Each node represents class (drug-trial) used for the AI training and testing procedure. Embedded line values represent AI prediction accuracy obtained from experimental testing runs following CNN training.

The present study used AI-driven approaches in three separate in-silico experiments based on SAB data collected in the Y-maze task described above. Experiment 1 merged both days into a single dataset, whereas Experiments 2 and 3 analyzed these days separately. In each experiment, we split data on the training and validation datasets and computed a confusion matrix for them, as in [34]. We identified data clusters within these matrices using the Louvain method and found that control fish and the 100-μg group belonged to the same cluster, whereas the 200-μg MPTP group formed a separate, distinct cluster regardless of the experimental day (Figure 4), linking the higher doses of MPTP to more significant changes in zebrafish locomotion.

## 4. Discussion

The present study established LD_50_ for intraperitoneal MPTP injection in adult zebrafish (~292 mg/kg) and applied AI-based data analysis of locomotion in the Y-maze test that further confirmed the 200-μg dose as that for which zebrafish behavioral patterns would cluster differently from control fish. Cognitive phenotypes were most prominently affected following the 200-μg MPTP treatment in zebrafish here, showing reduced SAB in the Y-maze test. Notably, the observed decline in SAB did not accompany locomotor deficits in zebrafish (Supplementary Material S1). 

Overall, the present study is the first report showing overt cognitive decline after MPTP exposure in adult zebrafish, detected without observing overt locomotor deficits. This finding raises two pertinent considerations: the validity of a behavioral endpoint as an indicator of fish cognition, and the potential influence of off-target drug effects. Indeed, the negative impact of MPTP on motor activity is well-documented in various animal studies [20,21], including those on zebrafish [37,38,39]. Specifically, MPTP-treated fish swim shorter distances, move more slowly, and freeze more often. The present PD model is therefore novel, as it revealed cognitive deficits in MPTP-treated fish without major decline in locomotor function.

To analyze the impact of MPTP on cognitive function more fully, we tested the effects of 200 μg MPTP on zebrafish associative learning and spatial working memory. The IAT was used to evaluate long-term associative memory formed via fear learning. As fear learning is evolutionarily conserved between fish and mammals [40], expectedly, control zebrafish displayed associative learning, as assessed by longer Day 2 deep-chamber (aversive stimulus) entry latency. Interestingly, no significant differences were observed for this endpoint in the drug-treated fish groups, suggestive of cognitive impairment due to MPTP action.

In contrast, SAB in the Y-maze differed in the 200-μg group from control fish, as supported by strong data clustering for this dose by AI-driven analysis. Interestingly, patients with advanced (late-stage) PD often demonstrate a loss in spatial and non-spatial working memory [41,42], whereas patients at the early stages display inattention, cognitive inflexibility, and executive dysfunction [41,43]. MPTP toxicity studies in primates report that chronic, low-dose MPTP impairs attention but not spatial working memory [44,45,46]. Likewise, MPTP-treated mice do not display loss of spatial working memory in the Y-maze, but do show worse associative learning in the passive avoidance tasks [47]. Moreover, the discrepancy between zebrafish and rodent responses to MPTP exposure may simply be the result of context, as, for example, MPTP-treated mice display poorer spatial working memory in Morris water maze [48]. Overall, our findings support PD-like cognitive decline observed in zebrafish following 200-μg MPTP treatment, paralleling cognitive deficits in PD models in mammals [49,50,51].

AI-driven analyses, especially based on artificial neural networks (ANNs), are becoming popular methods in animal behavioral analyses, including pioneering AI studies of zebrafish drug-induced behavior [34]. ANNs have also analyzed tracks of zebrafish in two [52] and three dimensions [53], as well as in larval fish [54]. More recently, unsupervised deep learning systems successfully linked zebrafish social behavior to dopamine D3 receptor agonism [55]. Aiming to further capitalize on this new powerful technology, here we incorporated machine learning to identifying locomotor patterns and MPTP dose in adult zebrafish.

AI analyses offer the advantage of reliably detecting complex changes in locomotion that may otherwise remain obscured during conventional statistical analysis. Increasingly, AI is employed in animal research to analyse multiple (from several to hundreds) individuals simultaneously while identifying novel effects of chemical agents as well as the latter’s interaction with each other. The adoption of AI technology in our study promotes the applications of this methodology and provides a point of reference for future researchers wishing to conduct high-throughput zebrafish drug experimentation.

In summary, intraperitoneal 200-μg MPTP induces PD-like cognitive, but not locomotor, deficits in adult zebrafish, hence strikingly paralleling specific late-stage cognitive PD symptoms in clinical patients. Co-application of AI-driven locomotor analyses and testing fish in well-established zebrafish cognitive assays further corroborates the validity of the proposed zebrafish model of late-stage PD. However, follow-up studies may be needed to further validate this zebrafish model by testing a wider range of MPTP doses (e.g., near the proposed LD_50_ dose), screening various pro- and anti-PD drugs, as well as characterizing genetic mutations in fish, relevant to clinical PD pathogenesis.

## Figures and Tables

**Table 1:** A summary of the design of in silico neural network Experiments 1–3 used in the present study. Experiment 1 included all data from both SAB testing days, Experiment 2A included data from Day 1 only, and Experiment 2B—data from Day 2 only. Experiment 3A used Day 1 data to train the neural network and Day 2 data to test it. Experiment 3B used data from Day 2 as a training dataset and Day 1 data for testing the network.

Datasets	Experiment 1	Experiment 2A	Experiment 2B	Experiment 3A	Experiment 3B
Day 1—Control	Training and testing	Training and testing		Training	Testing
Day 1—100 μg					
Day 1—200 μg					
Day 2—Control			Training and testing	Testing	Training
Day 2—100 μg					
Day 2—200 μg					

## Data Availability

Raw data from this study can be obtained from the corresponding author, upon reasonable request.

## Supplementary Materials

The following supporting information can be downloaded at: https://www.mdpi.com/article/10.3390/toxics10020069/s1, Figure S1: Zebrafish behaviors in the novel tank test; Table S1: The number of fish used in each of the two experiments.

## References

[1] Lee A., Gilbert R.M. (2016). Epidemiology of Parkinson disease. Neurol. Clin..

[2] Obeso J.A., Rodriguez-Oroz M.C., Rodriguez M., Lanciego J.L., Artieda J., Gonzalo N., Olanow C.W. (2000). Pathophysiology of the basal ganglia in Parkinson’s disease. Trends Neurosci..

[3] Mao Q., Qin W.Z., Zhang A., Ye N. (2020). Recent advances in dopaminergic strategies for the treatment of Parkinson’s disease. Acta Pharmacol. Sin..

[4] Secker D., Brown R. (2005). Cognitive behavioural therapy (CBT) for carers of patients with Parkinson’s disease: A preliminary randomised controlled trial. J. Neurol. Neurosurg. Psychiatry.

[5] Ramirez-Ruiz B., Junque C., Marti M.J., Valldeoriola F., Tolosa E. (2007). Cognitive changes in Parkinson’s disease patients with visual hallucinations. Dement. Geriatr. Cogn. Disord..

[6] Przedborski S., Jackson-Lewis V., Yokoyama R., Shibata T., Dawson V.L., Dawson T.M. (1996). Role of neuronal nitric oxide in 1-methyl-4-phenyl-1, 2, 3, 6-tetrahydropyridine (MPTP)-induced dopaminergic neurotoxicity. Proc. Natl. Acad. Sci. USA.

[7] Przedborski S., Jackson-Lewis V. (1998). Mechanisms of MPTP toxicity. Mov. Disord. Off. J. Mov. Disord. Soc..

[8] Przedborski S., Vila M. (2001). MPTP: A review of its mechanisms of neurotoxicity. Clin. Neurosci. Res..

[9] Langston J.W., Forno L.S., Rebert C.S., Irwin I. (1984). Selective nigral toxicity after systemic administration of 1-methyl-4-phenyl-1, 2, 5, 6-tetrahydropyrine (MPTP) in the squirrel monkey. Brain Res..

[10] Ballard P.A., Tetrud J.W., Langston J.W. (1985). Permanent human parkinsonism due to 1-methy 1-4-phenyl-1, 2, 3, 6-tetrahydropyridine (MPTP): Seven cases. Neurology.

[11] Beal M.F. (2001). Experimental models of Parkinson’s disease. Nat. Rev. Neurosci..

[12] Sallinen V., Torkko V., Sundvik M., Reenilä I., Khrustalyov D., Kaslin J., Panula P. (2009). MPTP and MPP+ target specific aminergic cell populations in larval zebrafish. J. Neurochem..

[13] Nicklas W.J., Vyas I., Heikkila R.E. (1985). Inhibition of NADH-linked oxidation in brain mitochondria by 1-methyl-4-phenyl-pyridine, a metabolite of the neurotoxin, 1-methyl-4-phenyl-1, 2, 5, 6-tetrahydropyridine. Life Sci..

[14] Berardo A., Musumeci O., Toscano A. (2011). Cardiological manifestations of mitochondrial respiratory chain disorders. Acta Myol..

[15] Schmidt D.E., Ebert M.H., Lynn J.C., Whetsell W.O. (1997). Attenuation of 1-methyl-4-phenylpyridinium (MPP+) neurotoxicity by deprenyl in organotypic canine substantia nigra cultures. J. Neural Transm..

[16] Meredith G.E., Rademacher D.J. (2011). MPTP mouse models of Parkinson’s disease: An update. J. Parkinson’s Dis..

[17] Razali K., Othman N., Nasir M.H.M., Doolaanea A.A., Kumar J., Ibrahim W.N., Ibrahim N.M., Mohamed W.M. (2021). The Promise of the Zebrafish Model for Parkinson’s Disease: Today’s Science and Tomorrow’s Treatment. Front. Genet..

[18] Parng C., Roy N.M., Ton C., Lin Y., McGrath P. (2007). Neurotoxicity assessment using zebrafish. J. Pharmacol. Toxicol. Methods.

[19] Rink E., Wullimann M.F. (2001). The teleostean (zebrafish) dopaminergic system ascending to the subpallium (striatum) is located in the basal diencephalon (posterior tuberculum). Brain Res..

[20] Jiang P.E., Lang Q.H., Yu Q.Y., Tang X.Y., Liu Q.Q., Li X.Y., Feng X.Z. (2019). Behavioral Assessments of Spontaneous Locomotion in a Murine MPTP-induced Parkinson’s Disease Model. J. Vis. Exp..

[21] Jenner P. (2003). The MPTP-treated primate as a model of motor complications in PD: Primate model of motor complications. Neurology.

[22] Selvaraj V., Venkatasubramanian H., Ilango K., Santhakumar K. (2019). A simple method to study motor and non-motor behaviors in adult zebrafish. J. Neurosci. Methods.

[23] Sallinen V., Kolehmainen J., Priyadarshini M., Toleikyte G., Chen Y.C., Panula P. (2010). Dopaminergic cell damage and vulnerability to MPTP in Pink1 knockdown zebrafish. Neurobiol. Dis..

[24] Lalonde R. (2002). The neurobiological basis of spontaneous alternation. Neurosci. Biobehav. Rev..

[25] Hughes R.N. (2004). The value of spontaneous alternation behavior (SAB) as a test of retention in pharmacological investigations of memory. Neurosci. Biobehav. Rev..

[26] Westerfield M. (2000). A Guide for the Laboratory Use of Zebrafish (Danio Rerio).

[27] de Abreu M.S., Giacomini A.C., Demin K.A., Petersen E.V., Kalueff A.V. (2021). On the value of zebrafish outbred strains in neurobehavioral research. Lab Anim..

[28] Serikuly N., Alpyshov E.T., Wang D.M., Wang J.T., Yang L.E., Hu G.J., Yan D.N., Demin K.A., Kolesnikova T.O., Galstyan D. (2021). Effects of acute and chronic arecoline in adult zebrafish: Anxiolytic-like activity, elevated brain monoamines and the potential role of microglia. Prog. Neuro Psychopharmacol. Biol. Psychiatry.

[29] Randhawa M.A. (2009). Calculation of LD_50_ values from the method of miller and Tainter, 1944. J. Ayub. Med. Coll. Abbottabad..

[30] Cognato G.D.P., Bortolotto J.W., Blazina A.R., Christoff R.R., Lara D.R., Vianna M.R., Bonan C.D. (2012). Y-Maze memory task in zebrafish (*Danio rerio*): The role of glutamatergic and cholinergic systems on the acquisition and consolidation periods. Neurobiol. Learn. Mem..

[31] Yamashita R., Nishio M., Do R.K.G., Togashi K. (2018). Convolutional neural networks: An overview and application in radiology. Insights Into Imaging.

[32] Lindsay G.W. (2020). Convolutional neural networks as a model of the visual system: Past, present, and future. J. Cogn. Neurosci..

[33] Liu M., Shi J., Li Z., Li C., Zhu J., Liu S. (2016). Towards better analysis of deep convolutional neural networks. IEEE Trans. Vis. Comput. Graph..

[34] Bozhko D.V., Myrov V.O., Kolchanova S.M., Polovian A.I., Galumov G.K., Demin K.A., Zabegalov K.N., Strekalova S.T., de Abreu M.S., Petersen E.V. (2022). Artificial intelligence-driven phenotyping of zebrafish psychoactive drug responses. Prog. Neuro Psychopharmacol. Biol. Psychiatry.

[35] Flach P. Performance evaluation in machine learning: The good, the bad, the ugly, and the way forward. Proceedings of the AAAI Conference on Artificial Intelligence.

[36] Que X., Checconi F., Petrini F., Gunnels J.A. Scalable community detection with the louvain algorithm. Proceedings of the 2015 IEEE International Parallel and Distributed Processing Symposium.

[37] Anichtchik O.V., Kaslin J., Peitsaro N., Scheinin M., Panula P. (2004). Neurochemical and behavioural changes in zebrafish Danio rerio after systemic administration of 6-hydroxydopamine and 1-methyl-4-phenyl-1, 2, 3, 6-tetrahydropyridine. J. Neurochem..

[38] Bretaud S., Lee S., Guo S. (2004). Sensitivity of zebrafish to environmental toxins implicated in Parkinson’s disease. Neurotox. Teratol..

[39] Sarath Babu N., Murthy C.L.N., Kakara S., Sharma R., Brahmendra Swamy C.V., Idris M.M. (2016). 1-Methyl-4-phenyl-1, 2, 3, 6-tetrahydropyridine induced Parkinson’s disease in zebrafish. Proteomics.

[40] Lal P., Tanabe H., Suster M.L., Ailani D., Kotani Y., Muto A., Itoh M., Iwasaki M., Wada H., Yaksi E. (2018). Identification of a neuronal population in the telencephalon essential for fear conditioning in zebrafish. BMC Biol.

[41] Owen A.M., Iddon J.L., Hodges J.R., Summers B.A., Robbins T.W. (1997). Spatial and non-spatial working memory at different stages of Parkinson’s disease. Neuropsychologia.

[42] Freedman M., Oscar-Berman M. (1986). Selective delayed response deficits in Parkinson’s and Alzheimer’s disease. Arch. Neurol..

[43] Cooper J.A., Sagar H.J., Jordan N., Harvey N.S., Sullivan E. (1991). Cognitive impairment in early, untreated Parkinson’s disease and its relationship to motor disability. Brain.

[44] Schneider J.S., Kovelowski C.J. II. (1990). Chronic exposure to low doses of MPTP. I. Cognitive deficits in motor asymptomatic monkeys. Brain Res..

[45] Roeltgen D.P., Schneider J.S. (1991). Chronic low-dose MPTP in nonhuman primates: A possible model for attention deficit disorder. J. Child Neurol..

[46] Decamp E., Tinker J.P., Schneider J.S. (2004). Attentional cueing reverses deficits in spatial working memory task performance in chronic low dose MPTP-treated monkeys. Behav. Brain Res..

[47] Moriguchi S., Yabuki Y., Fukunaga K. (2012). Reduced calcium/calmodulin-dependent protein kinase II activity in the hippocampus is associated with impaired cognitive function in MPTP-treated mice. J. Neurochem..

[48] Deguil J., Chavant F., Lafay-Chebassier C., Pérault-Pochat M.C., Fauconneau B., Pain S. (2010). Neuroprotective Effect of PACAP on Translational Control Alteration and Cognitive Decline in MPTP Parkinsonian Mice. Neurotox. Res..

[49] Kumar P., Kaundal R.K., More S., Sharma S.S. (2009). Beneficial effects of pioglitazone on cognitive impairment in MPTP model of Parkinson’s disease. Behav. Brain Res..

[50] Roeltgen D.P., Schneider J.S. (1994). Task persistence and learning ability in normal and chronic low dose MPTP-treated monkeys. Behav. Brain Res..

[51] Yan J., Liu A., Fan H., Qiao L., Wu J., Shen M., Lai X., Huang J. (2020). Simvastatin improves behavioral disorders and hippocampal inflammatory reaction by NMDA-mediated anti-inflammatory function in MPTP-treated mice. Cell. Mol. Neurobiol..

[52] Xu Z., Cheng X.E. (2017). Zebrafish tracking using convolutional neural networks. Sci. Rep..

[53] Pedersen M., Haurum J.B., Bengtson S.H., Moeslund T.B. 3D-ZeF: A 3D Zebrafish Tracking Benchmark Dataset. Proceedings of the 2020 IEEE/CVF Conference on Computer Vision and Pattern Recognition (CVPR).

[54] Wang X., Cheng E., Burnett I.S., Huang Y., Wlodkowic D. (2017). Automatic multiple zebrafish larvae tracking in unconstrained microscopic video conditions. Sci. Rep..

[55] Geng Y., Peterson R.T. (2021). Social behavioral profiling by unsupervised deep learning reveals a stimulative effect of dopamine D3 agonists on zebrafish sociality. bioRxiv.

