# Supplementary material for: MPTP-Treated Zebrafish Recapitulate ‘Late-Stage’ Parkinson’s-like Cognitive Decline
Source: Toxics. 2022 Feb 4;10(2):69. doi: 10.3390/toxics10020069 (PMC8879925; doi:10.3390/toxics10020069)
Supplement: Supplementary file 1 [file toxics-10-00069-s001.zip › toxics-1528844-supplementary.pdf]

toxics MDPI

Supplementary Materials: MPTP-treated zebrafish recapitulate 'late-stage' Parkinson's-like cognitive decline

Alim A. Bashirzade, Sergey V. Cherezis, Alisa S. Belova, Alexey V. Drobkov, Anastasiia D. Korotaeva, Soheil Azizi-Arani, Amirhossein Azimirad, Eric Odle, Emma-Yanina V. Gild, Oleg V. Ardashov, Konstantin P. Volcho, Dmitrii V. Bozhko, Vladislav O. Myrov, Sofia M. Kolchanova, Aleksander I. Polovian, Georgii K. Galumov, Nariman F. Salakhutdinov, Tamara G. Amstislavskaya and Allan V. Kalueff

Table S1. The number of fish used in each of the two experiments.

Group	Total fish	Fatalities	Mortality Rate (%)	Lethality of MPTP exposure (by dose), %
Control	27 + 12 = 39	5 + 1 = 6	15.38	
50 µg	12	3	25	9.7
100 µg	28 + 13 = 41	15+2 = 17	41.46	25.98
200 µg	25 + 16 = 41	15 + 8 = 23	56.09	40.72
400 µg	35	27	77.14	61.76

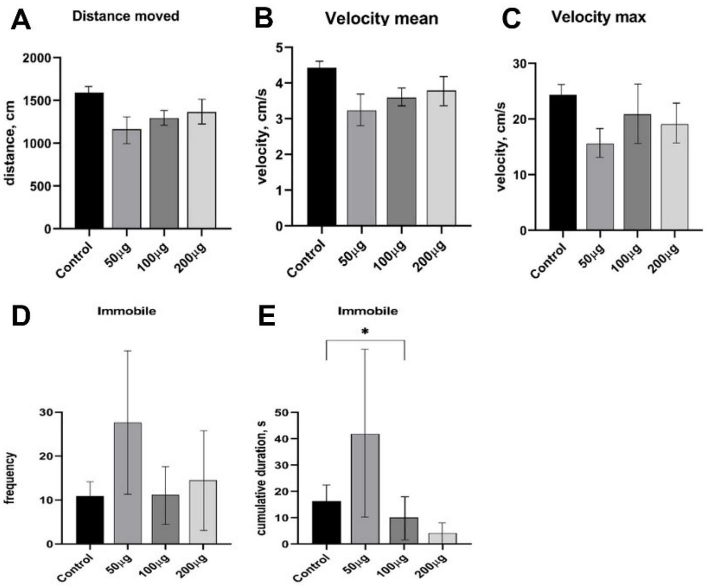

Figure S1. Zebrafish behaviors in the novel tank test. Data for panels (A) and (B) were analyzed using a one-way ANOVA (factor - dose) yielding no significant differences from vs. controls, n = 9-18 per group. Data for panels (C-E) were analyzed using Kruskal-Wallis H test. Data are presented as mean ± SEM. * - p < 0.05 vs Control fish, H test.
